# LUMEN-APPOSING METAL STENT AND ELETROCAUTERY ENHANCED DELIVERY SYSTEM (HOT AXIOSTM) FOR DRAINAGE OF WALLED-OFF NECROSIS: THE FIRST BRAZILIAN CASE REPORT

**DOI:** 10.1590/0102-672020180001e1430

**Published:** 2019-02-07

**Authors:** Diogo Turiani HOURNEAUX DE-MOURA, Galileu Ferreira Ayala FARIAS, Vitor Ottoboni BRUNALDI, Caio Vinicius TRANQUILLINI, Marcos Eduardo Lera DOS-SANTOS, Sérgio Eijii MATUGUMA, José JUKEMURA, Eduardo Guimarães HOURNEAUX DE-MOURA

**Affiliations:** 1 Digestive Endoscopy Service, Clinics Hospital, Medical School, University of São Paulo, São Paulo, SP, Brazil.

**Keywords:** Pancreatitis acute necrotizin, Pancreatic pseudocys, Endoscopy, Pancreatite necrosante agud, Pseudocisto pancreátic, Endoscopia

## INTRODUCTION

Pancreatic fluid collections are acute complications following pancreatitis^1^. They are usually self-limited and further interventions are only indicated for growing, symptomatic or complicated cases, such as infected or bleeding collections^2^. Currently, EUS-guided drainage is the gold-standard treatment^7^. The AXIOS is a lumen-apposing metal stent specifically developed for the treatment of pancreatic fluid collections. The juxtaposed lumen improved the limitations of others drainage devices. This characteristic reduces the migration and obstruction rates, and the necessity of stent substitutions. The Hot-AXIOS is an insulated delivery system plugged into the stent that allows transmural deployment of the AXIOS and exempts the guidewire placement and dilation of the tract^10^.

The objective of this study (Institutional Ethic Committee approval number 54011816.6.0000.0068) was to report the first Brazilian case of EUS-guided transmural drainage of a walled-off necrosis with the HOT-AXIOSTM system and to describe the technique for the deployment.

## TECHNIQUE

The initial steps to perform the procedure are: 1) wet the AXIOS catheter with sterile water or normal saline; 2) insert the AXIOS device system into the working channel; 3) connect the electrosurgical generator and set it to pure cut; 4) unlock the catheter lock; 5) advance the black catheter control hub until the catheter is seen by the ultrasound image; 6) energize the device and advance the catheter until the target structure; 7) lock the catheter when it is at 3 cm inside the target structure; 8) power off the generator and unplug the active cable from the AXIOS handle; 9) deploy the first flange taking off the yellow safety clip; 10) unlock the stent lock; 11) slide the stent deployment hub upwards until the stent deployment hub locks into place at the number 2 arrow.

To promote the alignment of the stent it´s necessary to unlock the catheter lock; to slide the catheter control hub upwards until at least 2 to 3 mm of the black catheter shaft marker is visible in the GI tract; finally, lock the catheter lock.

Next, is the deployment of the second flange made through unlocking the stent lock; sliding the stent deployment hub upwards towards the number 4 arrow; confirming that the second flange is deployed and visible in the GI tract, and finally, rotate counterclockwise the luer lock and remove the AXIOS delivery system.

## CASE REPORT

**FIGURE 1:**
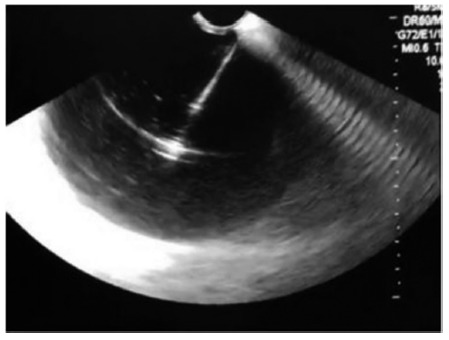
Puncture of the pancreatic cyst, predominantly anechoic, with some mobile heterogeneous material, consistent with walled-off necrosis

A 42-years-old alcoholic man was evaluated for a 30-day history of abdominal pain and postprandial vomiting. He referred two previous hospitalizations for acute pancreatitis. Physical examination was unremarkable. Laboratory studies revealed a slight increase of pancreatic enzymes. Magnetic resonance imaging showed a 13 cm collection with thick content in the pancreatic neck. The upper endoscopy found an important bulging in the posterior gastric wall and the endoscopic ultrasound revealed a 15.7x7.2 cm pancreatic cyst, predominantly anechoic, with some mobile heterogeneous material, consistent with walled-off necrosis (Figure 1).

**FIGURE 2:**
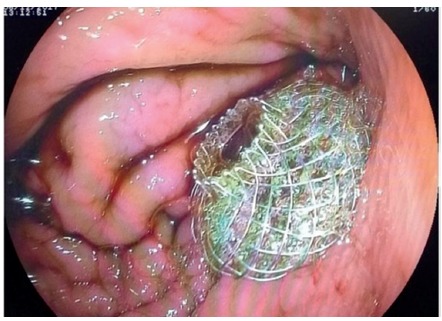
Endoscopic drainage with a 10x10 mm HOT-AXIOS^TM^ stent with an immediate output of a brownish secretion with necrotic solid components

Due to the diagnosis of symptomatic walled-off necrosis, we opted for an endoscopic drainage with a 10x10 mm HOT-AXIOS^TM^ stent (Figure 2). The patient was under general anesthesia. After puncture, there was an immediate output of a brownish secretion with necrotic solid components (Figure 2). The total duration of the procedure was 20 min and the time required for drainage was 4 min. We did not use fluoroscopy and there were no immediate complications.

The patient was discharged three days later, asymptomatic. On the 7^th^ post-drainage day, he underwent a new upper endoscopy and a pediatric endoscope was passed (5.4 mm in diameter) through the stent into the cyst cavity. Around 80% of the walled-off necrosis was collapsed and there was a small amount of fibrin with no clots or necrotic tissue. We washed it with 300 ml of 0.9% saline.

**FIGURE 3:**
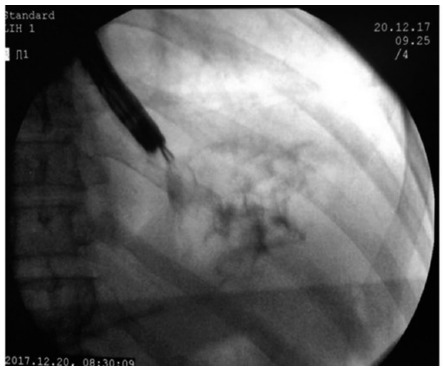
Hot-AXIOS^TM^ removed using a foreign body forceps

On the 14^th^ post-drainage day, we performed another upper endoscopy and an ERCP for assessment of the main pancreatic duct. Firstly, we tried to introduce a typical gastroscope through the stent but it was not possible due to the complete collapse of the prosthesis. Then, we instilled contrast through the stent and we notice complete reflux back to the gastric chamber, confirming the complete collapse of the collection. Pancreatography revealed a stricture in the transition of the head and neck but no fistula. We performed a pancreatic sphincterotomy and placed a 7Fx10 cm straight plastic stent through the stricture. After deployment of the pancreatic stent, we removed the Hot-AXIOS using a foreign body forceps (Figure 3). The patient was asymptomatic and was discharged 24 h after the ERCP procedure.

**FIGURE 4:**
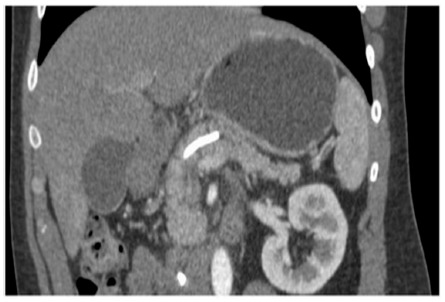
Abdominal CT showed complete resolution of the pancreatic fluid collection

On the 30^th^ post-drainage day, a new abdominal CT showed complete resolution of the pancreatic collection (Figure 4). The patient is currently on two months follow-up and remains asymptomatic. An ERCP is planned on the 3^rd^ month evaluation for removal of the pancreatic stent.

## DISCUSSION

Invasive procedures directed at pancreatic fluid collections are indicated for symptomatic, infected, or growing cysts, preferably at least four weeks after the onset^6^. Treatment can be performed by surgery, endoscopy or interventional radiology (percutaneous drainage). Surgical treatment is the most invasive modality and carries significant morbidity, whereas percutaneous drainage presents a higher incidence of fistulas and recurrence rates^3^. Currently, the endoscopic treatment is the gold-standard because it is less invasive while highly effective^7^. 

Stents with an internal diameter equal to or greater than 10 mm allow direct passage of the endoscope into the collection. However, we did not perform direct endoscopic necrosectomy in the first procedure due to the risk of migration. In our unit, we employ a step-up approach, that is, from the simplest to the most complex procedure according to the patient’s clinical response. Initially, we perform a simple drainage, followed by unclogging the stent with saline solution and hydrogen peroxide (if obstructed) and, ultimately, irrigation with a nasocystic drain. If all these steps fail, we indicate the direct endoscopic necrosectomy. Using a similar step-up approach, Nabi Z *et al* ^4^ diminished the need of the endoscopic necrosectomy from 40% to 10% of cases.

The Hot-AXIOS is an improvement of the standard AXIOS that carries an insulated delivery system plugged into the lumen-apposing metal stents, allowing cauterization and introduction of the stent simultaneously. This system exempts punctures, guidewire placement and dilation of the tract. Therefore, it reduces the risk of procedure-related complications such as fistulas, leaks to abdominal cavity and loss of the puncture during the exchange of devices^5^ ^,^ ^9^.

Walter *et al* ^8^ performed a multicenter prospective study enrolling 61 patients: 15 pancreatic pseudocysts and 46 walled-off necrosis. Technical success was achieved in 93% in the pancreatic pseudocysts group and in 81% of the remainder in the walled-off necrosis group. They performed the endoscopic necrosectomy in 59.6% of patients with it, and 19% of them required more than one procedure. Adverse events occurred in 9%. There was a detachment of the AXIOS stent in three cases during debridement and three spontaneous migration of the lumen-apposing metal stents. The stents were removed after a mean of 32 days after the procedure in 82% of the patients.

Despite being only recently available in our country and a more expensive device, it has already been proved effective in several countries. Further experiences with the Hot-AXIOS device in our unit will allow our personal evaluation of the real effectiveness and safety profile. In this first case using the new HOT-AXIOS device, we experienced technical and therapeutic success in a short period of time without any adverse events.
